# Supplementary figures and images for: Assessment and determinants of acute post-caesarean section pain in a tertiary facility in Ghana
Source: PLoS One. 2022 May 25;17(5):e0268947. doi: 10.1371/journal.pone.0268947 (PMC9132330; doi:10.1371/journal.pone.0268947)

**S1 Appendix Visual Analog Scale (VAS)**


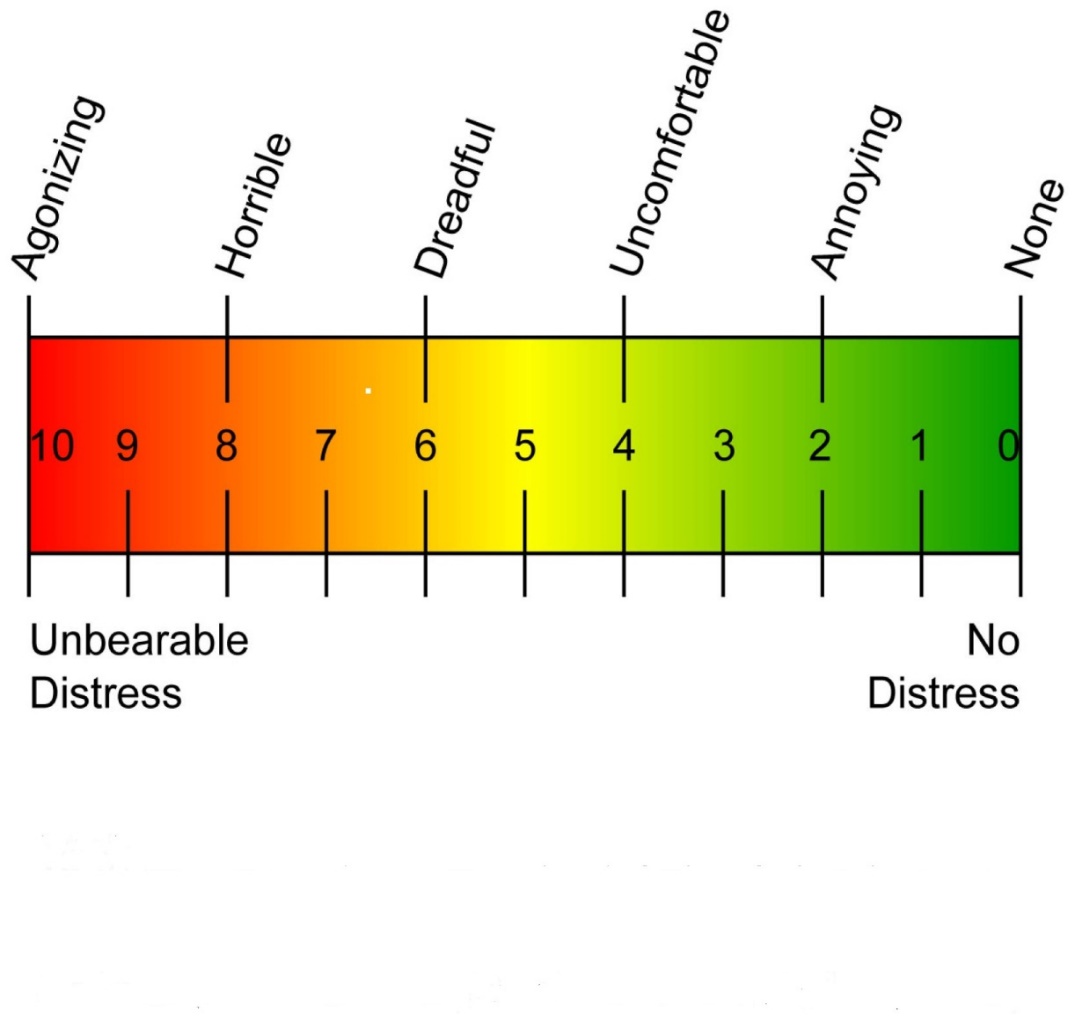

Supplement: S1 Appendix — (DOCX) [file pone.0268947.s001.docx]
