# Supplementary material for: Assessment and determinants of acute post-caesarean section pain in a tertiary facility in Ghana
Source: PLoS One. 2022 May 25;17(5):e0268947. doi: 10.1371/journal.pone.0268947 (PMC9132330; doi:10.1371/journal.pone.0268947)
Supplement: S2 Appendix — (DOCX) [file pone.0268947.s002.docx]

**S2 Appendix Stata bivariate and multivariate logistic regression analysis output**

1. **Logistic regression analysis; Marital status vs adequacy of pain at 6-12 hours post-CS at rest as outcome**

logistic painadequacy b1.MaritalStatus

Logistic regression Number of obs = 390

LR chi2(2) = 7.30

Prob > chi2 = 0.0260

Log likelihood = -266.65764 Pseudo R2 = 0.0135

------------------------------------------------------------------------------

painadequacy | Odds Ratio Std. Err. z P>|z| [95% Conf. Interval]

-------------+----------------------------------------------------------------

MaritalSta~s |

Married | 2.265233 .9446098 1.96 0.050 1.000366 5.129403

Co-habiting | 3.703704 1.851852 2.62 0.009 1.390066 9.868179

|

_cons | .45 .1806239 -1.99 0.047 .2049046 .9882648

------------------------------------------------------------------------------

1. **Logistic regression analysis; parity vs adequacy of pain at 6-12 hours post-CS at rest as outcome**

logistic painadequacy b1.paritygrp

Logistic regression Number of obs = 393

LR chi2(2) = 6.55

Prob > chi2 = 0.0379

Log likelihood = -269.12203 Pseudo R2 = 0.0120

------------------------------------------------------------------------------

painadequacy | Odds Ratio Std. Err. z P>|z| [95% Conf. Interval]

-------------+----------------------------------------------------------------

paritygrp |

para2 | .5218935 .149661 -2.27 0.023 .2974998 .9155395

multipara | .8964497 .2266623 -0.43 0.665 .5461405 1.471457

|

_cons | 1.238095 .2568572 1.03 0.303 .8244465 1.859284

------------------------------------------------------------------------------

1. **Logistic regression analysis; history of previous surgery vs adequacy of pain at 6-12 hours post-CS at rest as outcome**

logistic painadequacy b1. prevsg

Logistic regression Number of obs = 392

LR chi2(2) = 6.77

Prob > chi2 = 0.0338

Log likelihood = -268.28089 Pseudo R2 = 0.0125

------------------------------------------------------------------------------

painadequacy | Odds Ratio Std. Err. z P>|z| [95% Conf. Interval]

-------------+----------------------------------------------------------------

prevsg |

none | 1.844345 .4653796 2.43 0.015 1.124761 3.024294

at least .. | 1.231579 .3749699 0.68 0.494 .6781147 2.236771

|

_cons | .7037036 .149003 -1.66 0.097 .4646815 1.065673

------------------------------------------------------------------------------

1. **Logistic regression analysis; grade of surgeon vs adequacy of pain at 6-12 hours post-CS at rest as outcome**

logistic painadequacy b1.surgrade

Logistic regression Number of obs = 347

LR chi2(2) = 6.34

Prob > chi2 = 0.0420

Log likelihood = -234.68077 Pseudo R2 = 0.0133

------------------------------------------------------------------------------

painadequacy | Odds Ratio Std. Err. z P>|z| [95% Conf. Interval]

-------------+----------------------------------------------------------------

surgrade |

Resident | 2.748447 1.314861 2.11 0.035 1.076146 7.019456

Senior Re.. | 3.333333 1.653386 2.43 0.015 1.260867 8.812279

|

_cons | .4666667 .2136109 -1.67 0.096 .1902741 1.144548

------------------------------------------------------------------------------

1. **Logistic regression analysis; duration of caesarean section vs adequacy of pain at 6-12 hours post-CS at rest as outcome**

logistic painadequacy b1.duration

Logistic regression Number of obs = 389

LR chi2(1) = 28.73

Prob > chi2 = 0.0000

Log likelihood = -255.2392 Pseudo R2 = 0.0533

------------------------------------------------------------------------------

painadequacy | Odds Ratio Std. Err. z P>|z| [95% Conf. Interval]

-------------+----------------------------------------------------------------

duration |

more than.. | .3302518 .0695774 -5.26 0.000 .2185316 .4990869

_cons | 1.797101 .2699081 3.90 0.000 1.338844 2.412211

------------------------------------------------------------------------------

1. **Multivariate logistic regression analysis of marital status vrs adequacy of pain control at 6-12 hours post-CS (rest) adjusting for parity, history of previous surgery, duration of CS and grade of surgeon**

logistic painadequacy b1.MaritalStatus parity prevsg duration surgrade

Logistic regression Number of obs = 338

LR chi2(6) = 30.44

Prob > chi2 = 0.0000

Log likelihood = -217.14454 Pseudo R2 = 0.0655

------------------------------------------------------------------------------

painadequacy | Odds Ratio Std. Err. z P>|z| [95% Conf. Interval]

-------------+----------------------------------------------------------------

MaritalSta~s |

Married | 1.37328 .7216159 0.60 0.546 .4903197 3.846263

Co-habiting | 1.864017 1.102593 1.05 0.292 .5847253 5.942209

|

paritygrp | 1.213761 .1928798 1.22 0.223 .8889307 1.657289

prevsg | .7297323 .1184174 -1.94 0.052 .5309269 1.00298

duration | .3862901 .0932277 -3.94 0.000 .2407042 .6199312

surgrade | 1.313255 .2816579 1.27 0.204 .8625595 1.999442

_cons | 1.583864 1.248942 0.58 0.560 .3376839 7.428917

------------------------------------------------------------------------------

1. **Multivariate logistic regression analysis of parity vrs adequacy of pain control at 6-12 hours post-CS (rest) adjusting for marital status, history of previous surgery, duration of CS and grade of surgeon**

logistic painadequacy b1.paritygrp MaritalStatus prevsg surgrade duration

Logistic regression Number of obs = 338

LR chi2(6) = 34.29

Prob > chi2 = 0.0000

Log likelihood = -215.21898 Pseudo R2 = 0.0738

------------------------------------------------------------------------------

painadequacy | Odds Ratio Std. Err. z P>|z| [95% Conf. Interval]

-------------+----------------------------------------------------------------

paritygrp |

para2 | .6850903 .2278752 -1.14 0.256 .3569609 1.314846

multipara | 1.364 .4299874 0.98 0.325 .7353287 2.530155

|

MaritalSta~s |1.120561 .1273806 1.00 0.317 .8967565 1.400221

prevsg | .731829 .119588 -1.91 0.056 .5312697 1.008101

surgrade | 1.294054 .2774721 1.20 0.229 .8500355 1.970007

duration | .379689 .0921672 -3.99 0.000 .2359414 .6110152

_cons | 2.64532 1.966203 1.31 0.191 .6163115 11.35419

------------------------------------------------------------------------------

1. **Multivariate logistic regression analysis of history of previous surgery vrs adequacy of pain control at 6-12 hours post-CS (rest) adjusting for marital status, parity, duration of CS and grade of surgeon**

logistic painadequacy b0. prevsg MaritalStatus parity duration surgrade

Logistic regression Number of obs = 338

LR chi2(6) = 33.77

Prob > chi2 = 0.0000

Log likelihood = -215.48004 Pseudo R2 = 0.0727

------------------------------------------------------------------------------

painadequacy | Odds Ratio Std. Err. z P>|z| [95% Conf. Interval]

-------------+----------------------------------------------------------------

prevsg |

one previ.. | .4741991 .1343558 -2.63 0.008 .2721373 .8262918

at least .. | .6110164 .2034015 -1.48 0.139 .318197 1.173302

|

MaritalSta~s | 1.124543 .1279149 1.03 0.302 .8998149 1.405396

paritygrp | 1.187625 .1854228 1.10 0.271 .8745446 1.612786

duration | .4024086 .097663 -3.75 0.000 .2500829 .6475161

surgrade | 1.33285 .2846679 1.35 0.179 .8769662 2.02572

_cons | 1.759527 1.372911 0.72 0.469 .381265 8.120169

------------------------------------------------------------------------------

1. **Multivariate logistic regression analysis of grade of surgeon vrs adequacy of pain control at 6-12 hours post-CS (rest) adjusting for marital status, parity, duration of CS and history of previous surgery**

logistic painadequacy b1.surgrade MaritalStatus parity prevsg duration

Logistic regression Number of obs = 338

LR chi2(6) = 32.37

Prob > chi2 = 0.0000

Log likelihood = -216.17591 Pseudo R2 = 0.0697

------------------------------------------------------------------------------

painadequacy | Odds Ratio Std. Err. z P>|z| [95% Conf. Interval]

-------------+----------------------------------------------------------------

surgrade |

Resident | 2.505299 1.250674 1.84 0.066 .9417371 6.664833

Senior Re.. | 2.712934 1.438256 1.88 0.060 .9597908 7.668347

|

MaritalSta~s | 1.126608 .1275777 1.05 0.292 .902365 1.406576

paritygrp | 1.251491 .1933941 1.45 0.147 .924466 1.694201

prevsg | .7188804 .1168371 -2.03 0.042 .5227739 .9885517

duration | .3918102 .0947313 -3.88 0.000 .2439347 .6293294

_cons | 1.188043 .9039694 0.23 0.821 .2674004 5.278401

------------------------------------------------------------------------------

1. **Logistic regression analysis; marital status vrs adequacy of pain at 24-36 hours post-CS**

. logistic pnadq24_36 b1.MaritalStatus

Logistic regression Number of obs = 376

LR chi2(2) = 8.84

Prob > chi2 = 0.0120

Log likelihood = -150.29577 Pseudo R2 = 0.0286

-------------------------------------------------------------------------------

pnadq24_36 | Odds Ratio Std. Err. z P>|z| [95% Conf. Interval]

--------------+----------------------------------------------------------------

MaritalStatus |

Married | 3.119048 1.374835 2.58 0.010 1.314691 7.399807

Co-habiting | 7 5.066228 2.69 0.007 1.694508 28.91695

|

_cons | 2 .8164966 1.70 0.090 .8985214 4.451758

-------------------------------------------------------------------------------

1. **Multiple logistic regression; marital status vrs pain adequacy at 24-36 hours post-CS adjusting for parity, history of previous surgery, grade of surgeon and duration of CS**

logistic pnadq24_36 b1.MaritalStatus paritygrp prevsg surgrade duration

Logistic regression Number of obs = 327

LR chi2(6) = 7.08

Prob > chi2 = 0.3139

Log likelihood = -103.42206 Pseudo R2 = 0.0331

-------------------------------------------------------------------------------

pnadq24_36 | Odds Ratio Std. Err. z P>|z| [95% Conf. Interval]

--------------+----------------------------------------------------------------

MaritalStatus |

Married | 3.485438 2.33849 1.86 0.063 .9357495 12.98241

Co-habiting | 5.031581 4.285255 1.90 0.058 .9478952 26.70845

|

paritygrp | 1.059131 .26748 0.23 0.820 .6456267 1.737472

prevsg | 1.135589 .300561 0.48 0.631 .6759747 1.90771

surgrade | .5645311 .199941 -1.61 0.106 .2819786 1.130211

duration | .599366 .2364542 -1.30 0.194 .2766221 1.298665

_cons | 17.57012 21.70797 2.32 0.020 1.559952 197.8965

-------------------------------------------------------------------------------

1. **Backward elimination from (11) above; eliminating duration of CS**

logistic pnadq24_36 b1.MaritalStatus paritygrp prevsg surgrade

Logistic regression Number of obs = 330

LR chi2(5) = 5.39

Prob > chi2 = 0.3698

Log likelihood = -104.58058 Pseudo R2 = 0.0251

-------------------------------------------------------------------------------

pnadq24_36 | Odds Ratio Std. Err. z P>|z| [95% Conf. Interval]

--------------+----------------------------------------------------------------

MaritalStatus |

Married | 3.500617 2.337488 1.88 0.061 .945729 12.95754

Co-habiting | 5.244549 4.458879 1.95 0.051 .9908834 27.75836

|

paritygrp | 1.058597 .2692212 0.22 0.823 .6430636 1.742638

prevsg | 1.047998 .2686364 0.18 0.855 .6341174 1.732014

surgrade | .6326962 .217859 -1.33 0.184 .3221791 1.242491

_cons | 6.635191 6.425173 1.95 0.051 .994476 44.27031

-------------------------------------------------------------------------------

1. **Eliminating grade of surgeon from (12) above**

logistic pnadq24_36 b1.MaritalStatus paritygrp prevsg

Logistic regression Number of obs = 372

LR chi2(4) = 9.58

Prob > chi2 = 0.0482

Log likelihood = -149.30246 Pseudo R2 = 0.0311

-------------------------------------------------------------------------------

pnadq24_36 | Odds Ratio Std. Err. z P>|z| [95% Conf. Interval]

--------------+----------------------------------------------------------------

MaritalStatus |

Married | 3.257539 1.50736 2.55 0.011 1.315265 8.068001

Co-habiting | 7.582235 5.516886 2.78 0.005 1.821618 31.56002

|

paritygrp | .9648659 .1949124 -0.18 0.859 .6494054 1.433567

prevsg | 1.106319 .220735 0.51 0.613 .7482508 1.635739

_cons | 1.919294 1.02126 1.23 0.220 .6764186 5.445871

-------------------------------------------------------------------------------

1. **Eliminating history of previous surgery from (13) above**

logistic pnadq24_36 b1.MaritalStatus paritygrp

Logistic regression Number of obs = 372

LR chi2(3) = 9.32

Prob > chi2 = 0.0253

Log likelihood = -149.43176 Pseudo R2 = 0.0302

-------------------------------------------------------------------------------

pnadq24_36 | Odds Ratio Std. Err. z P>|z| [95% Conf. Interval]

--------------+----------------------------------------------------------------

MaritalStatus |

Married | 3.278477 1.516111 2.57 0.010 1.324462 8.115304

Co-habiting | 7.409831 5.379851 2.76 0.006 1.785665 30.74799

|

paritygrp | .9935983 .1930099 -0.03 0.974 .6789878 1.453984

_cons | 1.910009 1.016299 1.22 0.224 .6731602 5.419415

-------------------------------------------------------------------------------
