# Supplementary material for: Assessment and determinants of acute post-caesarean section pain in a tertiary facility in Ghana
Source: PLoS One. 2022 May 25;17(5):e0268947. doi: 10.1371/journal.pone.0268947 (PMC9132330; doi:10.1371/journal.pone.0268947)
Supplement: Attachment — Submitted filename: 2ND REVIEWER RESPONSE.docx [file pone.0268947.s004.docx]

**RESPONSE TO ACADEMIC REVIEWER (2^ND^ RESUBMISSION)**

1. Thank you the submission of your revised manuscript to PLOS ONE and for your attention to the journals requests.

We appreciate that you have sought verbal consent from the parents of the minors included in the study. Please could you clarify whether the IRB have specifically approved for verbal parental consent as the method of obtaining informed consent in this case.

**Response:** The Ethics Committee (Committee for Human Research and Publication Ethic as indicated in the manuscript) specifically approved parental/guardian informed consent for the case of participants under 18 years of age.

The relevant section in the current revised and resubmitted manuscript (lines 176-179) reads as follows;

“For participants below 18 years of age (one aged 14, two aged 16 and three aged 17), assent was documented but a verbal consent was obtained from the parent or guardian. Verbal parental/guardian informed consent for such cases was specifically approved by the ethics committee before commencement of the study.”
